# Phosphorylation of LCRMP-1 by GSK3β Promotes Filopoda Formation, Migration and Invasion Abilities in Lung Cancer Cells

**DOI:** 10.1371/journal.pone.0031689

**Published:** 2012-02-21

**Authors:** Wen-Lung Wang, Tse-Ming Hong, Yih-Leong Chang, Chen-Tu Wu, Szu-Hua Pan, Pan-Chyr Yang

**Affiliations:** 1 Graduate Institute of Life Sciences, National Defense Medical Center, Taipei, Taiwan; 2 Institute of Biomedical Sciences, Academia Sinica, Taipei, Taiwan; 3 Graduate Institute of Clinical Medicine, National Cheng Kong University, Tainan, Taiwan; 4 Department of Pathology and Graduate Institute of Pathology, National Taiwan University, Taipei, Taiwan; 5 Graduate Institute of Clinical Genomics, National Taiwan University College of Medicine, Taipei, Taiwan; 6 Department of Internal Medicine, National Taiwan University College of Medicine, Taipei, Taiwan; 7 NTU Center of Genomic Medicine, National Taiwan University, Taipei, Taiwan; Emory University, United States of America

## Abstract

LCRMP-1, a novel isoform of CRMP-1, can promote cancer cell migration, invasion and associate with poor clinical outcome in patients with non-small-cell lung cancer (NSCLC). However, the underlying regulatory mechanisms of LCRMP-1 in cancer cell invasiveness still remain obscure. Here, we report that GSK3β can phosphorylate LCRMP-1 at Thr-628 in consensus sequences and this phosphorylation is crucial for function of LCRMP-1 to promote filopodia formation, migration and invasion in cancer cells. Impediment of Thr-628 phosphorylation attenuates the stimulatory effects of LCRMP-1 on filopodia forming, migration and invasion abilities in cancer cells; simultaneously, kinase-dead GSK3β diminishes regulation of LCRMP-1 on cancer cell invasion. Furthermore, we also found that patients with low-level Ser-9-phosphorylated GSK3β expression and high-level LCRMP-1 expression have worse overall survival than those with high-level inactive GSK3β expressions and low-level LCRMP-1 expressions (P<0.0001). Collectively, these results demonstrate that GSK3β-dependent phosphorylation of LCRMP-1 provides an important mechanism for regulation of LCRMP-1 on cancer cell invasiveness and clinical outcome.

## Introduction

Metastasis contributes to treatment failure and death in the majority of cancer patients [1]. The capacity of cancer cells to progressive metastasis is controlled by complicated cellular processes, involving microenvironmental changes, increasing ability of cell migration or invasion, multiple genetic events and regulatory factors. Until now, many master inducers and suppressors of cancer metastasis has been identified to be involved in these processes, and thus unraveling upstream regulatory pathways of these proteins may facilitate depicting detailed molecular mechanisms for cancer metastasis [2].

Glycogen synthase kinase-3β (GSK3β) is known as a multi-tasking serine/threonine kinase that control numerous cellular processes including glycogen metabolism, cell differentiation, apoptosis, cytoskeleton rearrangement, cell cycle regulation, and cell proliferation [3], [4]. GSK3β regulates a broad range of substrates through phosphorylation at optimal consensus motifs (Ser/Thr-X-X-X-Ser/Thr, where X is representative of any amino acid) [3], [5]. Usually, most common substrates of GSK3β need a specific priming kinase to increase the efficiency of first phosphorylation at serine or threonine residues that near to the four residues of GSK3β phosphorylation site in the carboxyl terminus. For example, casein kinase 1 prior primes β-catenin to GSK3β phosphorylation [6], and casein kinase 2 is a priming kinase of glycogen synthase [7].

Collapsin response mediator protein-1 (CRMP-1) suppresses neuronal growth cone extension during development, and is also known as a cancer invasion suppressor [8], [9]. Recently, we identified a novel isoform of CRMP-1, the long form CRMP-1 (LCRMP-1) [10]. LCRMP-1 can promote filopodia formation, cancer cell migration, invasion through functionally against CRMP-1, and its expression correlates with poor clinical outcome in non-small-cell lung cancer (NSCLC) patients. LCRMP-1 and CRMP-1 harbors identical C-terminus sequences from exon-2 to exon-14; however, N-terminal exon-1 sequence of LCRMP-1 is distinct with that of CRMP-1 [11]. Among human CRMP family, amino acid sequence of CRMP-2 is 78% and 76% identity with CRMP-1 and CRMP-3, respectively [12]. Previously, CRMP-2 has been reported to be phosphorylated by GSK3β at Thr-514, and associated with impairing neuronal polarization [13]. Notably, CRMP-1 and CRMP-3 showed highly similar GSK3β phosphorylation consensus motifs with CRMP-2 [14]. Consistent with CRMP-1, LCRMP-1 also contain same motif for GSK3β phosphorylation.

Since LCRMP-1 and CRMP-1 have opposite function on cancer migration and invasion, whether the function of LCRMP-1 may be regulated by GSK3β should be further studied. In the present report, we investigate possible regulation of GSK3β on LCRMP-1. Here, we demonstrated that GSK3β can phosphorylate LCRMP-1 and modulate filopodia formation, cancer cell migration and invasion. We further confirm the GSK3β-phosphorylated site in LCRMP-1, investigate its function for cell invasiveness and evaluate its clinical significant in NSCLC patients.

## Results

### GSK3β can phosphorylate LCRMP-1 at Thr-628

**Figure 1:**
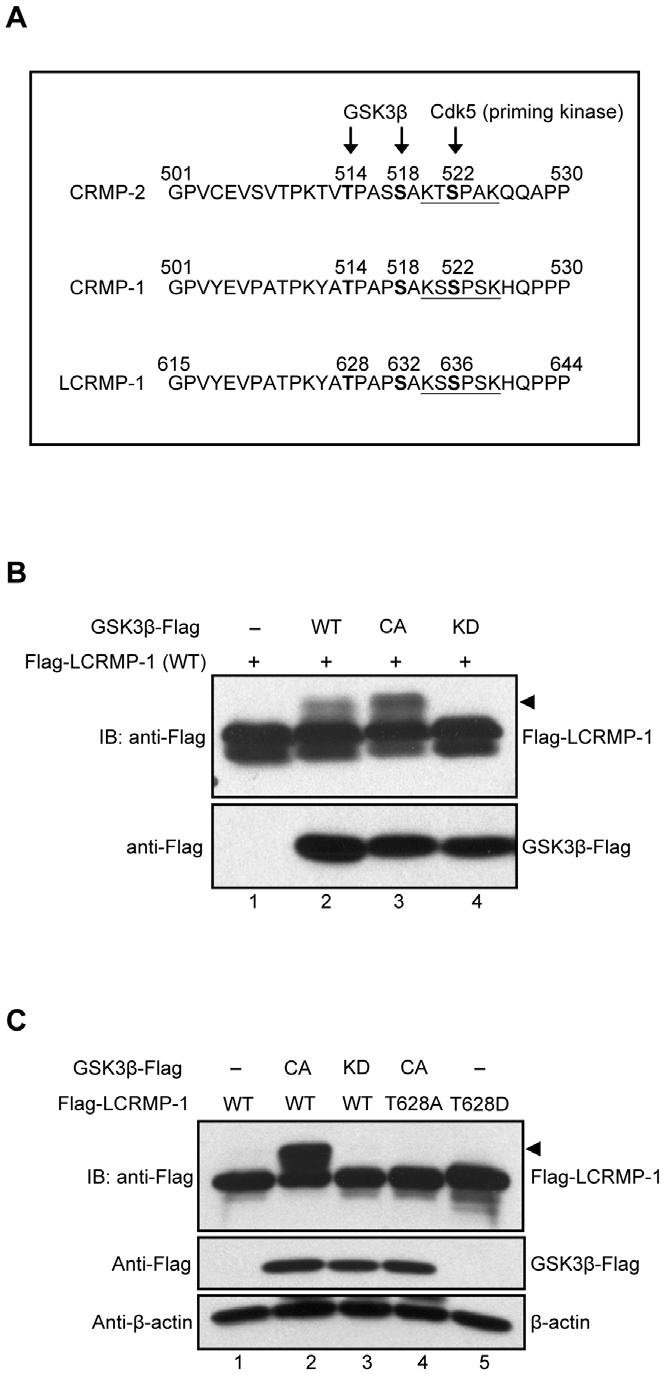
LCRMP-1 is a substrate of and phosphorylated by GSK3β at Thr-628. (A) Protein sequence analysis showed the potential consensus site of LCRMP-1 for the phosphorylation by GSK3β. Protein sequences are aligned among CRMP2, CRMP-1, and LCRMP-1. The numbers represent amino acid sites and underline shows a potential phosphorylation site for Cdk5. (B) GSK3β phosphorylates LCRMP-1 (WT) *in vivo*. HEK293T cells were co-transfected with Flag-tagged LCRMP-1 and distinct activity of Flag-tagged GSK3β (WT, CA and KD form). Equal amount of plasmids were transfected into every condition by using empty vectors. Cells were lysed 30 hr post-transfection and protein extracts were analyzed by immunoblotting with anti-Flag antibodies. (C) GSK3β phosphorylates LCRMP-1 (WT) at Thr-628 *in vivo*. CL1-0 cells were co-transfected either Flag-tagged LCRMP-1(WT) or LCRMP-1 (T628A, T628D) mutants in the presence or absence of distinct activity of Flag-tagged GSK3β (CA and KD form). Empty vectors were used for supplement to equal amount of plasmids in transfection assay. Cell lysates were harvested 48 hr post-transfection and analyzed by immunoblotting with anti-Flag and anti-β-actin antibodies.

To predict whether the classic GSK3β phosphorylation consensus sequences are existed in LCRMP-1, we first aligned the protein sequences among CRMP-2, CRMP-1, and LCRMP-1 (Fig. 1A). Previous study showed that Cdk5 is a priming kinase that phosphorylates CRMP-2 at Ser-522, following with phosphorylation of CRMP-2 at Thr-514 by GSK3β and resulting in functional regulation of neuronal polarization [13]. Therefore, we found that protein sequences of LCRMP-1 contained highly consistent with Cdk5 and GSK3β phosphorylation motif, thus we speculated that a major potential phosphorylation site of LCRMP-1 is located at Thr-628 (Fig. 1A). To examine whether LCRMP-1 can be phosphorylated by GSK3β, HEK293T cells were cotransfected with wild-type Flag-tagged LCRMP-1 (WT) in the presence of empty vector, wild-type GSK3β (WT), constitutively active GSK3β (CA), or kinase-dead GSK3β (KD). Expression of GSK3β (CA) was more obviously detected mobility shifts (arrowheads) of LCRMP-1 (WT) than GSK3β (WT) (Fig. 1B, lane 2 and 3). However, slow-migration upper bands were completely disappeared in cells expressing kinase-deficient form of GSK3β (KD) (Fig. 1B, lane 4). These results suggest that LCRMP-1 is a substrate of GSK3β and slowly migrating bands were caused by its phosphorylation *in vivo*.

Next, to determine whether GSK3β phosphorylates LCRMP-1 at Thr-628 *in vivo*, a nonphosphorylated LCRMP-1 mutant was generated by replacing Thr-628 to Ala (T628A). CL1-0 cells were cotransfected with LCRMP-1 (WT) or a LCRMP-1 (T628A) nonphosphorylated mutant in the presence of either empty vector, GSK3β (CA), or GSK3β (KD). Consistent with previous observations, GSK3β (CA) and GSK3β (KD) were proved to display band shift and non-band shift (arrowheads) of LCRMP-1, respectively (Fig. 1C, lane 2 and 3). Notably, LCRMP-1 (T628A) was resistant to GSK3β (CA) activity and the shifted bands were almost abolished (Fig. 1C, lane 4). This result was similar to the conditions of LCRMP-1 (WT) plus GSK3β (KD) (Fig. 1C, lane 3 and 4), and further confirming that GSK3β indeed phosphorylated LCRMP-1 at Thr-628 residues. Collectively, all these results indicated that LCRMP-1 was specifically phosphorylated at Thr-628 by GSK3β *in vivo*.

### Thr-628 phosphorylation of LCRMP-1 is required for cancer cell invasion, migration and filopodia formation

**Figure 2:**
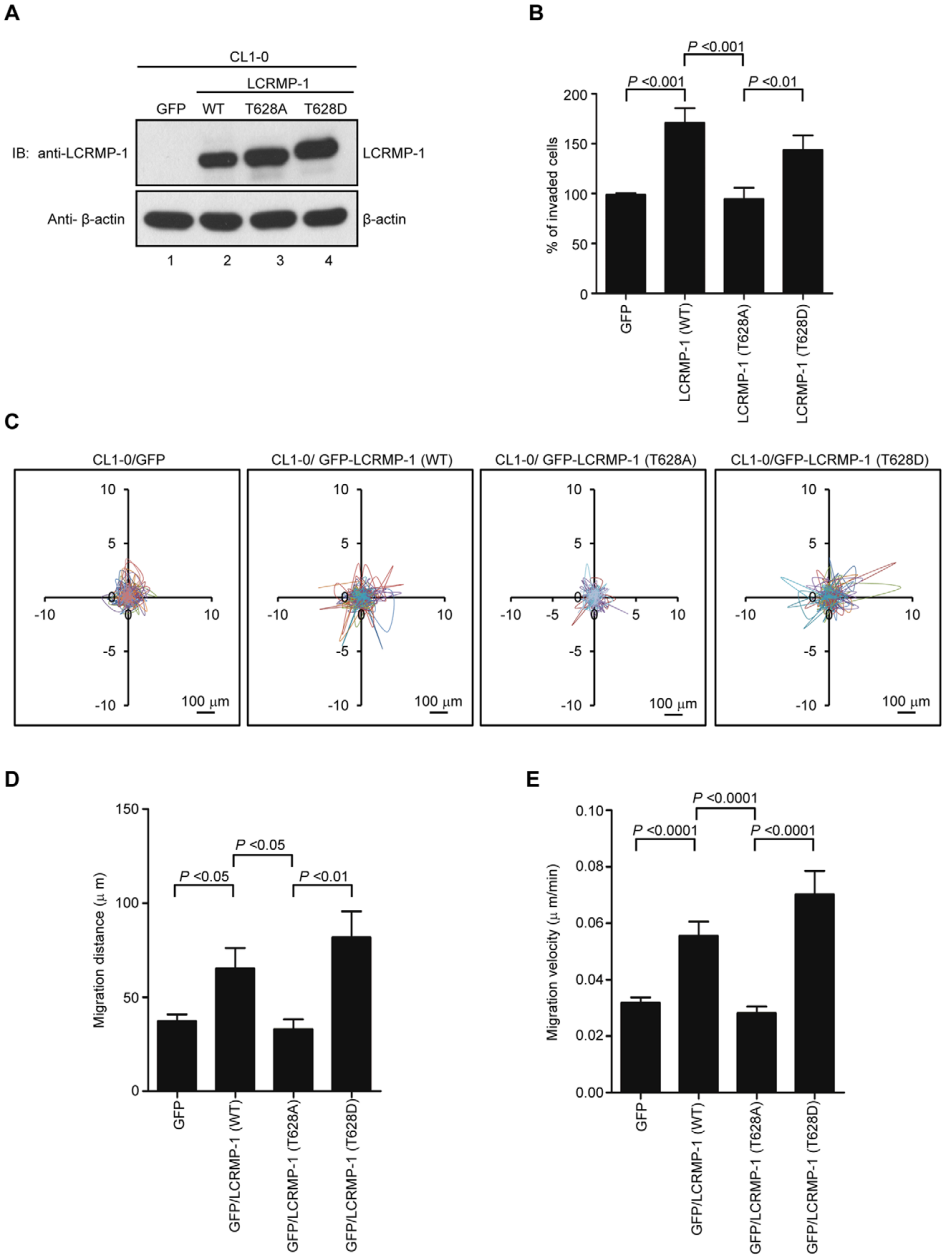
Phosphorylation of LCRMP-1 at Thr-628 is critical for cancer invasion and migration. (A) Protein expression levels of exogenous untagged LCRMP-1 (WT), LCRMP-1 (T628A), and LCRMP-1 (T628D) are confirmed by immunoblotting. After 48 hours post lentivirus infection, CL1-0 cells were stably expressing wild-type and mutant LCRMP-1. Lentivirus expressing GFP served as control. Cell lysates were harvested, following by assessing with immunoblotting using anti-LCRMP-1 antibody and anti-β-actin antibodies. (B) Nonphosphorylated LCRMP-1 (T628A) mutant lowers activity of cell invasion. The invasive capacity of these cells was determined with the modified Boyden chambers invasion assay *in vitro*. Percentage of invasive ability was normalized to GFP control. Data were shown as means ± SEM for three-independent experiments (n = 3). (C) Nonphosphorylated LCRMP-1 (T628A) mutant greatly suppressed cell migration tracks. Tract plots showed CL1-0 cells expressing GFP, GFP-LCRMP-1 (WT), GFP-LCRMP-1 (T628A), GFP-LCRMP-1 (T628D), respectively. Moving tracks of at least 10 representative cells at the start point all set to ‘0,0’ over a 20-hour period (different lines) showed the representative motility of cells. Scale bar, 100 µm. (D, E) Total migration distance (D) and cell migration velocity (E) were quantified from cell tracking assay for 20 hours. Data were presented as means ± SEM.

In our current reports, we have found that wild-type LCRMP-1 enhances filopodia formation, and promotes cell migration and invasion in noninvasive human lung cancer cell lines [10]. Since LCRMP-1 can be phosphorylated at Thr-628 by GSK3β, we next questioned whether the function of LCRMP-1 could be regulated by this phosphorylation. To address this, we generated a series of lentivirus that express GFP (control), non-tagged LCRMP-1 (WT), LCRMP-1 (T628A), or LCRMP-1 (T628D), and transduced them into low-invasive CL1-0 lung cancer cells which express low level of endogenous LCRMP-1 [11]. Protein expression of wild-type or mutant LCRMP-1 was confirmed by immunoblotting analysis using anti-LCRMP-1 antibodies (Fig. 2A). These cells were then used to examine the ability of cell invasion. As expected, LCRMP-1 (WT) overexpression contributed to an increased cell invasiveness compared with GFP control (Fig. 2B). However, T628A nonphosphorylated mutant of LCRMP-1 was greatly diminished cell invasiveness (Fig. 2B). Conversely, phospho-mimic LCRMP-1 (T628D), which was expected to mimic the phosphorylated form, displayed enhanced invasion ability similar to wild-type LCRMP-1 (WT). To further explore the effect of Thr-628 phosphorylation of LCRMP-1 expression on CL1-0 cell motility, we performed video time-lapse microscopy assay to monitor moving tracks of at least 10 individual cells over a 20-hour period. Lentivirus-transduced CL1-0 cells expressing GFP-LCRMP-1 (WT) or GFP-LCRMP-1 (T628D) increased both migration distance and migration velocity compared to GFP vector (Fig. 2C, D, and E). However, GFP-LCRMP-1 (T628A) showed marked compression of distance and velocity of migration (Fig. 2C, D, and E). These results suggested that phosphorylation of LCRMP-1 at Thr-628 is a prerequisite for cell invasiveness and cell migration.

**Figure 3:**
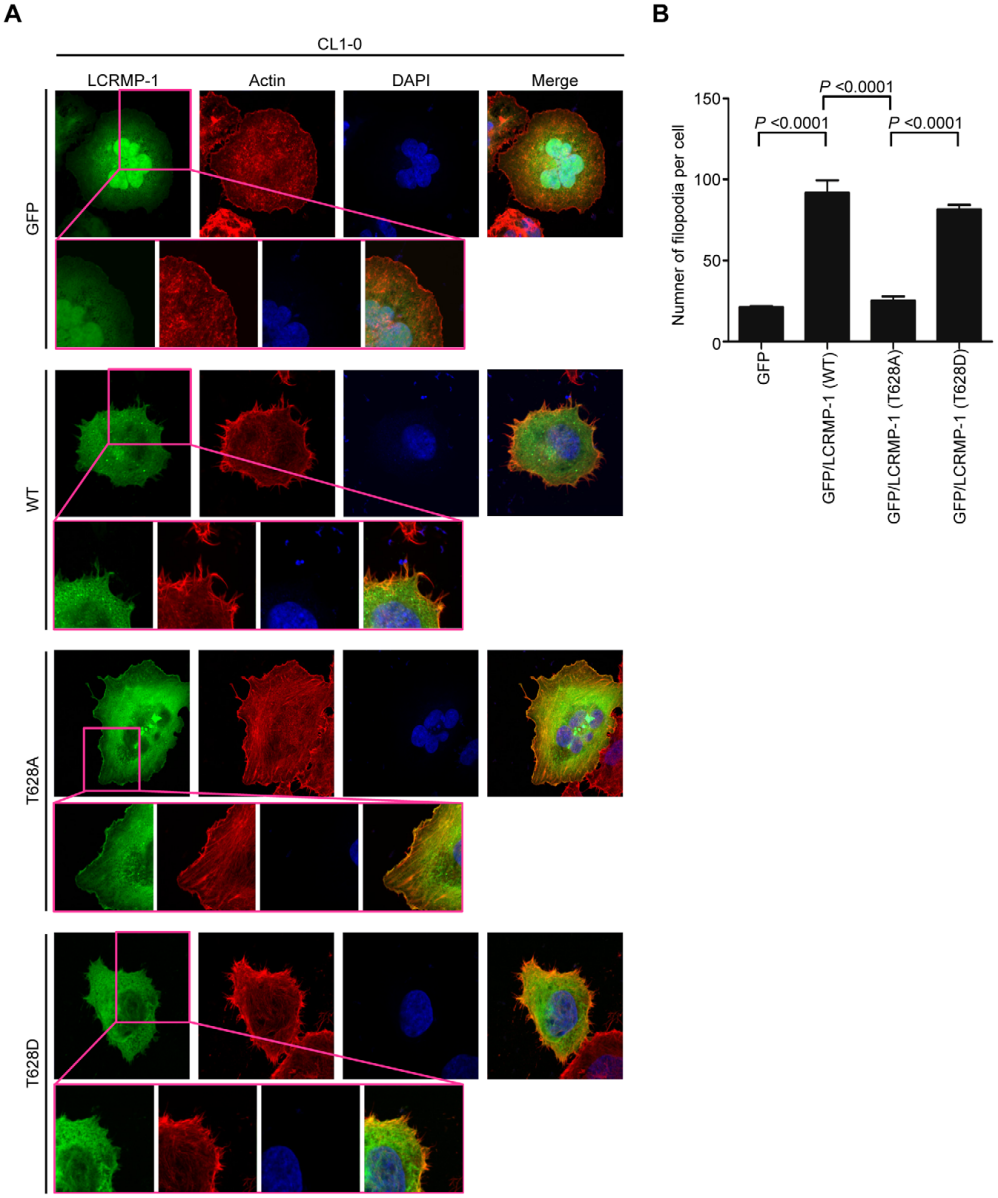
Phosphorylation of LCRMP-1 at Thr-628 is required for filopodia formation. (A) Nonphosphorylated LCRMP-1 (T628A) mutant impairs filopodia formation. CL1-0 cells were transiently transfected with GFP-tagged LCRMP-1 (WT), LCRMP-1 (T628A), and LCRMP-1 (T628D). At 24 hours after transfection, these cells were fixed, permeabilized and then immunostained with rhodamine-conjugated phalloidin (red) for actin and DAPI (blue) for visualizing nuclei. Representative immunofluorescence images were visualized using fluorescence microscope. Scale bar, 10 µm. Insets show higher magnifications (400×). (B) Numbers of filopodia were counted with at least six cells per group. Data were presented as means ± SEM.

Next, we further examined the effects of GSK3β on LCRMP-1 induced filopodia formation. CL1-0 cells were transiently transfected with GFP control, GFP-LCRMP-1 (WT), GFP-LCRMP-1 (T628A), or GFP-LCRMP-1 (T628D) and following by staining with actin using rhodamine-conjugated phalloidin. Consistent with our current reports, immunofluorescence analysis revealed that numbers of filopodia that induced by ectopic expression GFP-LCRMP-1 (WT) in CL1-0 cells were more than that by GFP vector control. Conversely, numbers of filopodia formation were obviously attenuated in cells expressing nonphosphorylated mutant GFP-LCRMP-1 (T628A) (Fig. 3A; 3B, p<0.0001). In addition, GFP-LCRMP-1 (T628D) was also induced more filopodia that similar to GFP-LCRMP-1 (WT), (Fig. 3A; 3B, p<0.0001). These data indicated that phosphorylation of LCRMP-1 at Thr-628 is crucial for filopodia formation.

### GSK3β phosphorylates LCRMP-1 and modulates cancer cell invasion

**Figure 4:**
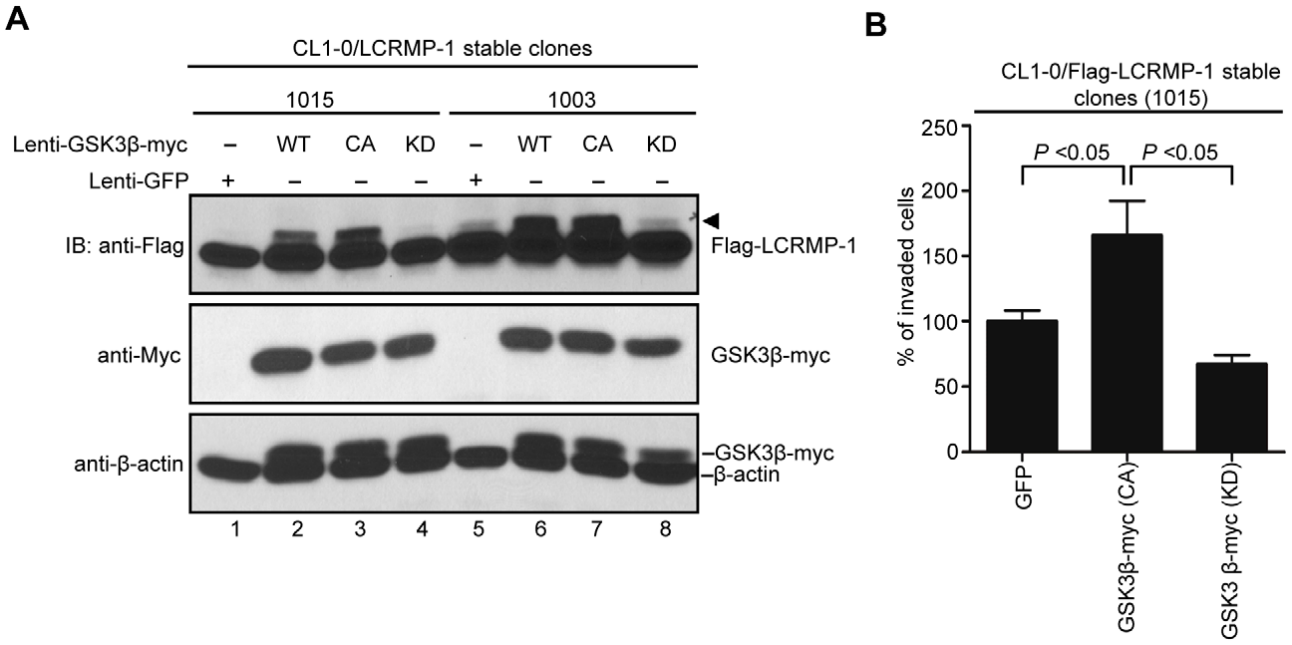
GSK3β modulates ability of LCRMP-1-induced cancer cell invasion. (A) Lentivirus expressed GFP control, myc-tagged GSK3β (WT), GSK3β (CA), or GSK3β (KD) in CL1-0/LCRMP-1 (WT) overexpression cells (1015 and 1003). After 48 hours postinfection, these cells were lysed and subjected to immunoblotting analysis with using anti-Flag, anti-Myc, and anti-β-actin antibodies. (B) GSK3β activity affects LCRMP-1-induced cancer cell invasion. CL1-0/LCRMP-1 overexpression cells (1015) were infected with lentivirus expressing GFP control, myc-tagged GSK3β (CA) or GSK3β (KD). After 48 hours postinfection, these cells were subjected to the modified Boyden chambers invasion assay *in vitro*. Normalization to GFP control served as percentage of invasive ability.

To further detect the effects of GSK3β on LCRMP-1(WT)-induced cancer cell invasion, lentivirus expressing GFP control, GSK3β (WT), GSK3β (CA), or GSK3β (KD) were infected in CL1-0/LCRMP-1 overexpression cells (lines 1015 and 1003) which have been previously shown to strongly induce cell invasiveness [10]. Consistent with our previous findings (Fig. 1B and C), immunoblotting analysis showed that GSK3β (CA) induced LCRMP-1 with shifted band compared to GFP control (Fig. 4A, lane 1 and 3; lane 5 and 7), but a non-shifted band was observed in GSK3β (KD) (Fig. 4A, lane 4 and lane 8). Based on above conditions, the results of invasion assay were also shown that GSK3β (CA)-introduced CL1-0/LCRMP-1 cells (1015) could further promote cell invasion compared to control cells (Fig. 4B). However, GSK3β (KD)-introduced cells resulted in a decreased in invasion ability (Fig. 4B). Taken together, these results demonstrated that GSK3β could modulate LCRMP-1 activity through a phosphorylation-dependent manner to control cancer cell invasion.

### Low expression of inactive GSK3β and high expression of LCRMP-1 correlate with poor overall survival in NSCLC patients

**Figure 5:**
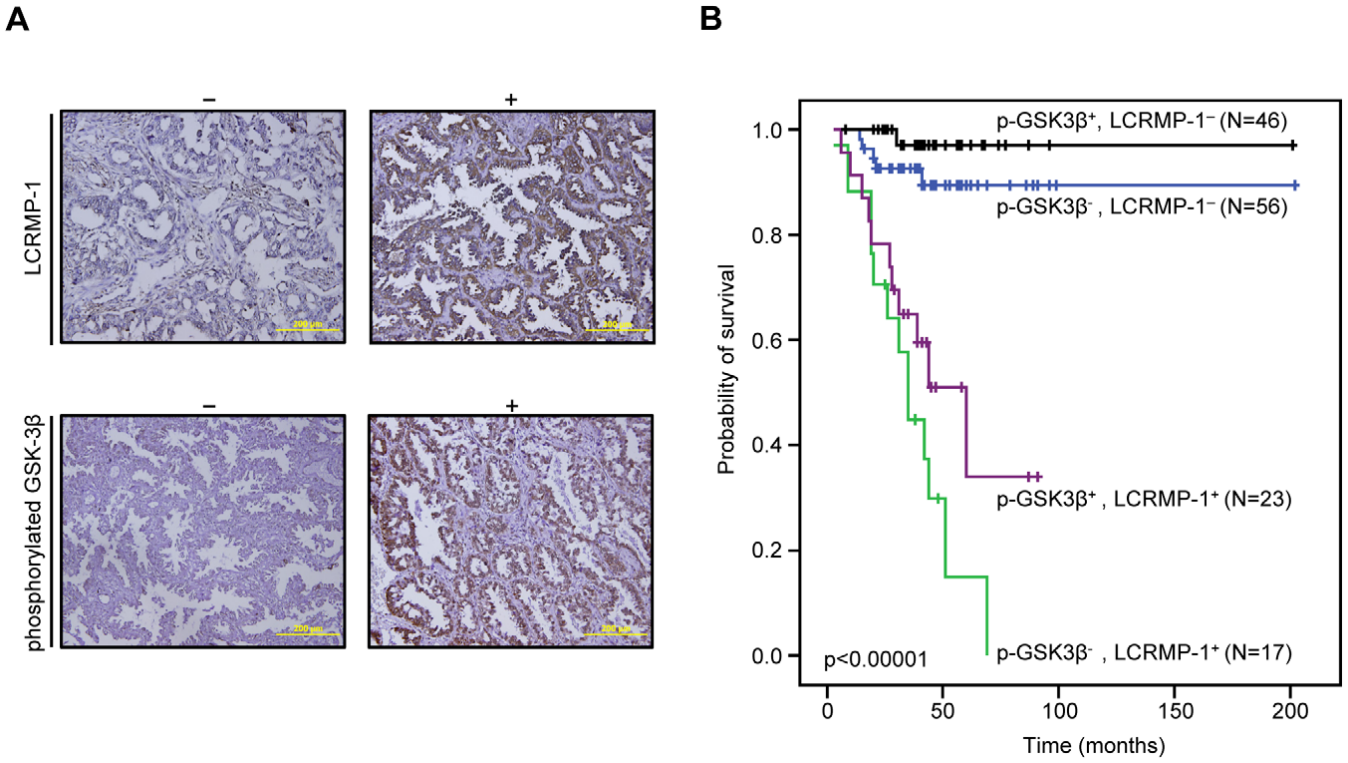
Kaplan-Meier survival plots for NSCLC patients grouped by phosphorylated GSK3β and LCRMP-1 protein expression levels. (A) Typical protein expression patterns of phosphorylated GSK3β and LCRMP-1 were detected by immunohistochemistry using anti-phospho-GSK3β (Ser9) and anti-LCRMP-1 antibodies (C2) in serial dissections of primary tumor specimens from 142 NSCLC patients who underwent surgical resections. Results are shown + and − denotes tumors with and without over-expression with indicated protein respectively. Scale bars, 100 µm. p-GSK3β was represented to phosphorylated GSK3β. (B) Kaplan–Meier analysis of overall survival for 142 NSCLC patients with p-GSK3β^−^-LCRMP-1^−^, p-GSK3β^−^-LCRMP-1^+^, p-GSK3β^+^-LCRMP-1^−^, and p-GSK3β^+^-LCRMP-1^+^. *P* values were performed by 2-sided log-rank tests.

Although our results consistently suggested that function of LCRMP-1 could be regulated by GSK3β phosphorylation, such studies do not fully reflect clinical malignancy. Accordingly, we extended our analysis by examining inactive form GSK3β and LCRMP-1 protein expression levels in tumor specimens from 142 NSCLC patients. The clinical characteristics of these patients are summarized in Table 1. Serial sections of each specimen were stained with antibodies against LCRMP-1 and Ser-9-phosphorylated GSK3β that indicated the status of inactive form GSK3β due to the Akt-mediated activation which results in suppression of GSK3β activity through phosphorylation at Ser-9 [15]. Our results showed typical staining of LCRMP-1 and Ser-9-phosphorylated GSK3β in patient's specimen (Fig. 5A). Consistent with our previous reports, high-level LCRMP-1 had significantly poor overall survival compared with low-level LCRMP-1 in patients with NSCLC [10], [11]. Notably, analysis of the combined effect of both proteins on patients' prognoses revealed that patients with low-level expression of inactive form GSK3β and high-level expression of LCRMP-1 had poorer overall survival than those with high-level inactive form GSK3β expression and low-level LCRMP-1 expression (Fig. 5B, p<0.00001). Multivariable Cox proportional-hazards regression analyses, with a stepwise selection model, were present to evaluate the associations of various independent prognostic factors with patient survival (Table 2). These results suggest that high activity GSK3β and high-level LCRMP-1, possibly mimicking the phosphorylated status of LCRMP-1, are associated with increasing cancer invasiveness and poorer overall survival.

**Table 1:** LCRMP-1 and phosphorylated GSK3β expression in relation to clinical parameters and pathological characteristics*.

			LCRMP-1	p-GSK3β				
Category	Subcategory	Number	≤50% (%)	>50% (%)	*P*	≤70% (%)	>70% (%)	*P*
Total patients		142	102 (71.8)	40 (28.2)		73 (51.4)	69 (48.6)	
Sex	Female	78	55 (53.9)	23 (57.5)	0.7	44 (60.3)	34 (49.3)	0.188
	Male	64	47 (46.1)	17 (42.5)		29 (39.7)	35 (50.7)	
Histological type†	Adenocarcinoma	123	86 (87.8)	37 (94.9)	0.215	60 (85.7)	63 (94.0)	0.108
	Squamous cell carcinoma	14	12 (12.2)	2 (5.1)		10 (14.3)	4 (6.0)	
Tumor size, cm	>3	74	57 (55.9)	17 (42.5)	0.151	41 (56.2)	33 (47.8)	0.32
	≤3	68	45 (44.1)	23 (57.5)		32 (43.8)	36 (52.2)	
Vascular invasion	Positive	25	19 (18.6)	6 (15.0)	0.61	14 (19.2)	11 (15.9)	0.613
	Negative	117	83 (81.4)	34 (85.0)		59 (80.8)	58 (84.1)	
Lymph node metastasis	Positive	32	18 (17.7)	14 (35.0)	0.026	18 (24.7)	14 (20.3)	0.534
	Negative	110	84 (82.4)	26 (65.0)		55 (75.3)	55 (79.7)	
Extranodal extension	Positive	20	10 (9.8)	10 (25.0)	0.019	11 (15.1)	9 (13.0)	0.729
	Negative	122	92 (90.2)	30 (75.0)		62 (84.9)	60 (87.0)	
Tumor stage	Stage I	107	82 (80.4)	25 (62.5)		53 (72.6)	54 (78.3)	
	Stage II	17	13 (12.8)	4 (10.0)		11 (15.1)	6 (8.7)	
	Stage III–IV	18	7 (6.9)	11 (27.5)	0.004	9 (12.3)	9 (13.0)	0.505
LCRMP-1 expression, %	>50	102	–	–	–	56 (76.7)	46 (66.7)	0.184
	≤50	40	–	–		17 (23.3)	23 (33.3)	
p-GSK3β expression, %	>70	69	46 (45.1)	23 (57.5)	0.184	–	–	–
	≤70	73	56 (54.9)	17 (42.5)		–	–	

*P values were calculated using a two-sided chi-squared test. Abbreviations: LCRMP-1, long-form collapsin response mediator protein-1; p-GSK3β, phosphorylated Glycogen synthase kinase-3β.

† Adenosquamous carcinomas are not included.

**Table 2:** Hazard ratios for death (from any cause) among patients with NSCLC, according to multivariable Cox regression analysis*.

Variable	Hazard ratio (95% C.I.)	*P*
LCRMP-1	26.22 (7.56 to 90.94)	<.0001
p-GSK3β	0.48 (0.2 to 1.16)	0.102
Sex	2.48 (0.97 to 6.37)	0.058
Histological type	0.15 (0.04 to 0.52)	0.003
Tumor stage	0.47 (0.3 to 0.73)	0.001

*Stepwise selection was used to choose the optimal multivariable Cox proportional hazard regression model. LCRMP-1 and phosphorylated GSK3β expression was designated as ‘high’ or ‘low’ using 50% and 70% cell positivity as the cut-off point respectively, and were adjusted by histological type (squamous cell carcinoma as the referent vs. adenocarcinoma), and stage (stage I as the referent vs. stage II vs. stage III). *P* values (two-sided) were calculated using a chi-square test. Abbreviations, LCRMP-1, long-form collapsin response mediator protein-1; p-GSK3β, phosphorylated Glycogen synthase kinase-3β; CI, confidence interval.

## Discussion

Our study primarily investigated the regulatory mechanism of post-translation modification associated with the cancer cell migration and invasiveness of LCRMP-1. Here, we showed that GSK3β-dependent phosphorylation of LCRMP-1 positively regulates filopodia formation, migration and cancer cell invasion. On the basis of GSK3β-phosphorylated consensus motifs, Thr-628 amino acid residue of LCRMP-1 is the master phosphorylation site for GSK3β (Fig. 1). A substitution of Thr-628 for Ala in LCRMP-1 led to impair filopodia formation, migration and cancer cell invasion, whereas a replacement of Thr-628 to Asp greatly restored its function (Fig. 2 and 3). Consistent with these observations, ectopic expression of kinase-dead GSK3β diminished LCRMP-1-induced invasive ability (Fig. 4). Moreover, clinical NSCLC patients with low-level of inactive GSK3β and high-level LCRMP-1 protein expression is associated with poor overall survival than those with high-level inactive form GSK3β expression and low-level LCRMP-1 expression (Fig. 5B). Thus, our results provide evidence to support the crucial mechanism of GSK3β-dependent phosphorylation to control the LCRMP-1-mediated filopodia formation, migration and invasive abilities in cancer cells.

Sequence analysis indicated that there has a Cdk5 (priming kinase) phosphorylation site in both LCRMP-1 and CRMP-1 (Fig. 1A). After phosphorylation at Ser-636 by Cdk5, GSK3β in turn phosphorylates Ser-632 and Thr-628 sequentially. Therefore, GSK3β may induce slower migrating bands in LCRMP-1 including both Ser-632 and Thr-628 phosphorylation in cells, and the bands induced by constitutively active GSK3β were more active than that of wild-type GSK3β (Fig. 1B). In detailed analysis, we found that LCRMP-1 mutant, T628A, could block most of GSK3β phosphorylation induced migrating bands (Fig. 1C). This may indicate that Thr-628 of LCRMP-1 may be the dominant and important phosphorylation site for GSK3β phosphorylation. However, we could not exclude the possibility that constitutively active GSK3β can phosphorylate other sites of LCRMP-1.

The process of tumor invasion is primarily through alternations of the extracellular matrix including actin polymerization and filopodia formation [16]. Our findings reveal that blockage of GSK3β-mediated phosphorylation of LCRMP-1 at Thr-628 leads to a regression of filopodia formation. A previous report was described that GSK-3 phosphorylation for Paxillin is necessary for cytoskeletal rearrangement [17]. Thus, we speculate that GSK3β may positively promote regulation of actin cytoskeleton and tumor invasion. In contrast to positively role, GSK3β is also reported to perform its suppressive roles for its substrates [18]. GSK3β phosphorylation for some nuclear transcription factors, such as β-catenin and Snail, trigger proteasomal degradation, following with suppression on epithelial–mesenchymal transition (EMT) and tumor invasion [6], [19]–[21]. GSK3β simultaneously localizes in cytoplasm and nucleus [22], as consistent with our results of immunohistochemical staining (Fig. 5A), and LCRMP-1 is a stable cytosolic protein [10]. Therefore, GSK3β govern cell invasion is possibly dependent on the characterization of its protein substrates. High-level LCRMP-1 expression is associated with poor overall and disease-free survival compared to low expression group in NSCLC patients [10], [11]. Thus, LCRMP-1 potentially serves as a best candidate to identify high-risk patients. In this study, we showed a very interesting finding that patients with low-level phosphorylated GSK3β and high-level LCRMP-1 expressions had worse overall survival than the other catalog groups. Focusing on the low-level LCRMP-1 expression or the high-level LCRMP-1 expression group, we could also found that high-level of phosphorylated GSK3β expression may discriminate better outcome from low-level phosphorylated GSK3β expression, respectively. The combined effects of inactive form GSK3β and LCRMP-1 protein expression may have important clinical implications to indicate the high-risk subset of NSCLC patients as candidates for additional effective adjuvant therapy. The results suggest that GSK3β phosphorylation of LCRMP-1 is associated with poor clinical outcome. However, there still have some limitations in our experiments. Although the reference indicated that Ser-9-phosphorylated GSK3β can indicate the status of inactive form GSK3β due to the Akt-mediated activation which results in suppression of GSK3β activity through phosphorylation at Ser-9 [15], whether Ser-9 phosphorylation of GSK3β inhibits its ability to phosphorylate LCRMP-1 is still not clear. To solve this problem, generating a specific anti-phospho-Thr-628 LCRMP-1 antibody for immunohistochemistry should be the most important issues in the future. This may confirm the clinical significance of GSK3β induced phosphorylation of LCRMP-1 in NSCLC patients.

In addition, we also found that patients with low-levels (n = 73) or high-levels (n = 69) of Ser-9-phosphorylated GSK3β expression were almost equal in distribution. Cells exist at different actual levels of active GSK3β unless distinct signaling pathways for inhibiting GSK3β are triggered concurrently, such as MAPK and phosphatidylinositol-3-OH kinase/Akt pathways [20]. Thus, we speculate that distinct activated extent of signaling pathways to induce an inhibition of GSK3β activity may lead to different outcome for patients with LCRMP-1 expression. Therefore, further investigating upstream signaling pathway for regulation of GSK3β may provide a better diagnosis for NSCLC patients with low-levels or high-levels of LCRMP-1 expression.

In conclusion, we show a new regulatory mechanism for GSK3β to phosphorylate an invasion enhancer LCRMP-1 and thus could further fine-tune cancer cell invasion abilities. Additionally, LCRMP-1 expression and Ser-9-phosphorylated GSK3β levels may have clinical implications in the outcome prediction of patients with NSCLC.

## Materials and Methods

### Ethics statement

This investigation was approved by the Institutional Review Board of the National Taiwan University Hospital and obtained informed written consent statement from all participant patients involved in our study.

### Patients and Tumor Specimens

Lung tumor tissue specimens were obtained from patients (n = 142) with histologically confirmed NSCLC who had undergone complete surgical resections at the National Taiwan University Hospital (Taipei, Taiwan) between December 28, 1995, and December 26, 2005. This investigation was approved by the Institutional Review Board of the National Taiwan University Hospital. The enrolled patients had not been treated with neoadjuvant chemotherapy or irradiation therapy. All specimens were formalin fixed, sectioned, stained with hematoxylin and eosin, and examined by microscopy. Pathological staging was performed by Dr. Yih-Leong Chang (Department of Pathology and Graduate Institute of Pathology, National Taiwan University) according to the international staging system for lung cancer [23].

### Immunohistochemical analysis

Immunohistochemical staining of tumor tissue samples from patients with NSCLC was carried out as previously described [11]. In brief, the sections for analysis of LCRMP-1 or phosphorylated GSK3β protein expression were first autoclaved in Trilogy Solution (Cell Marque Corp., Rocklin, CA.) or Antigen Retrieval Citra Solution (Biogenex, San Ramon, CA) at 121°C for 10 minutes. The samples were subsequently made a treatment of 3% H_2_O_2_-methanol, incubation with DakoCytomation Dual EndogenousEnzyme Block (DakoCytomation, Inc., Carpinteria, CA) for 10 minutes, Ultra V Block (Lab Vision Corporation, Fremont, CA) for 10 minutes, antibody-dilution buffer (Ventana Medical Systems, Inc., Tucson, AZ) for 10 minutes, and finally with a phosphorylated GSK3β (Cell signaling, Danvers, MA) antibody for 6 hours at room temperature or a polyclonal anti–LCRMP-1 antibody (C2; 1∶300 dilution) overnight at 4°C. Detection of the immunostaining was determined using Super Sensitive Non-Biotin Polymer HRP Detection System (BioGenex, San Ramon, CA) according to the manufacturer's protocol.

### Modified Boyden chamber invasion assay

Modified Boyden chambers with polycarbonate-membrane inserts (pore size 8 µm; Falcon, Becton Dickinson) coated with 30 µg Matrigel (BD) were performed cell invasion assays. 2.5×10^4^ cells suspended in RPMI medium containing 10% NuSerum (Invitrogen, Eugene, OR) were plated in the upper chambers, and 1 ml medium was added to cover the lower chambers. After 24 hours incubation at 37°C, cells were fixed with methanol at room temperature for 10 minutes. After fixation, samples were stained with a 50 µg/ml solution of propidium iodide (Sigma, St. Louis, MO) at room temperature for 30 minutes. Each membrane was photographed and counted the number of cells under a microscope at a magnification of ×50, using the Analytical Imaging Station software package (Imaging Research Inc., St. Catharines, ON, Canada). Each experiment was assayed in triplicate.

### Immunofluorescence staining for observation of filopodia formation

Transfected or lentivirus-infected cells were fixed with 3.7% cold paraformaldehyde, washed with PBS, following by permeabilizing with 0.1% Triton X-100. The cells were then stained with rhodamine-conjugated phalloidin (red, Molecular Probes, Eugene, OR). The cells were mounted onto microscope slides with ProLong® Gold antifade reagent with DAPI (Molecular Probes) and then examined and photographed using LSM 700 laser scanning confocal microscope from Carl Zeiss.

### Cell migration analysis

Moving tracks of migrating cells were performed by video time-lapse microscopy as previously described [24]. In brief, cells were maintained in growth medium at 37°C/5% CO_2_ and time-lapse images were observed under a AF 6000 LX microscope (Meyer Instruments,Inc.) for the time period of 20 hours. Images were taken with a CoolSNAP HQ CCD camera (Roper Scientific, NJ) at 5-minute intervals and processed by MetaMorph 5.0 software (Universal Imaging, Downingtown, PA).

### Cell culture and transfection

The human lung adenocarcinoma cell lines (CL1-0 cells) were isolated from a 64-year-old male patient with a poorly differentiated adenocarcinoma and selected in our laboratory by in vitro Transwell invasion to get 5 sublines with progressive invasiveness, with similar genotypic background (designated CL1-1, CL1-2, CL1-3, CL1-4, and CL1-5) as previously described [25]. HEK293T cell lines were purchased from American Type Culture Collection (ATCC, USA). The CL1-0 and HEK293T cells were grown in RPMI and DMEM medium containing 10% FBS and 2 mM L-glutamine (all from Invitrogen, Eugene, OR) at 37°C in a humidified atmosphere of 5% CO_2_-95% air, respectively. All cell lines in this study were tested with mycoplasma-free condition. All transfection experiments were carried out using Lipofectamine or Lipofectamine 2000 reagents (Invitrogen) according to the manufacturer's instructions.

### Protein sequences alignment and Plasmids

Amino acid sequences alignment of CRMP-2, CRMP-1, and LCRMP-1 are based on their Gene bank accession number NP_001377, NP_001304, and NP_001014809, respectively. The LCRMP-1 expression plasmid pCMV-Tag2A-LCRMP-1(WT) and pEGFP-LCRMP-1(WT) were described as previously [10]. Amino-acid substitution mutants of LCRMP-1 were generated by PCR-based site-directed mutagenesis with a QuikChange kit (Stratagene, Santa Clara, CA) and verified by DNA sequencing. FLAG-tagged GSK3β (WT, CA and KD form) expression plasmids were subcloned from pCMV-5A-GSK3β (WT, CA and KD form, a gift from M.-C. Hung) into pFLAG -CMV-5a vector (Sigma).

### Antibodies

Primary antibodies for immunoblotting were as follows: monoclonal anti-Flag (M2; Sigma), anti-Myc (9E11; Millipore, Billerica, MA), anti-β-actin (Sigma) and polyclonal anti-LCRMP-1 antibody. The HRP conjugated goat anti-mouse and goat anti-rabbit secondary antibodies were purchased from (Amersham Biosciences, Pittsburgh, PA).

### Lentivirus production and transduction

GFP-tagged, untagged LCRMP-1 (WT, T628A and T628D), and myc-tagged GSK3β (WT, CA and KD form) were constructed by cloning their cDNA into pTYEF lentiviral vector. Briefly, HEK293T cells were contransfected with the indicated lentiviral vector and three helper plasmids pHP-dl-N/A, pHEF-VSVG, and pCEP4-Tat by using Lipofectamine 2000 reagents according to manufacturer's protocols. Virus-containing medium was collected at 24, 48, 72 hours post-transfection, centrifuged, and filtered through 0.45 µm-pore-size filters. The percentage of pTYEF-GFP-infected cells by flow cytometry were determined the relative lentivirus titers. Cells were infected with GFP or the indicated lentivirus in media containing polybrene (8 µg/ml). After twenty-four hours post-infection, cells were treated with fresh medium for 24–48 hours and then used for all experiments.

### Cell lysate preparation and immunoblotting

All experiments were performed according to standard protocols. Briefly, preparation of whole-cell lysates for immunoblotting and immunoprecipitation were using IP lysis buffer (20 mM Tris, pH 7.5, 150 mM NaCl, 0.5% Nonidet P-40, 100 µM Na_3_VO_4_, 50 mM NaF, 30 mM sodium pyrophosphate) containing protease inhibitors (protease inhibitor cocktail; Roche Diagnostics, Basel, Switzerland). After brief sonication and centrifugation, protein samples were resolved by SDS-PAGE gels, transferred into PVDF membranes (Millipore), blotted with the indicated antibodies and finally detected chemiluminescent signals using X-ray films.

### Statistical analysis

Data are shown as Mean ± SEM. and statistical analyses were performed by Student's *t*-test or Pearson's *χ*2 test. The overall survivals for patient groups with different expression signatures were determined using SPSS software (v10.0; SPSS, Inc., Chicago, IL) by the Kaplan–Meier method and two-sided log-rank tests. Immunoreactivity in more than 50% and 70% of the tumor specimens was defined to high level of LCRMP-1 and phosphorylated GSK3β expression, respectively. P values<0.05 were considered to be statistically significant.
